# Linewidth narrowing in self-injection-locked on-chip lasers

**DOI:** 10.1038/s41377-023-01172-9

**Published:** 2023-06-28

**Authors:** Emad Alkhazraji, Weng W. Chow, Frédéric Grillot, John E. Bowers, Yating Wan

**Affiliations:** 1 Integrated Photonics Lab, King Abdullah University of Science and Technology, Thuwal, 23955 Saudi Arabia; 2 Sandia National Laboratories, Albuquerque, NM 87185-1086 USA; 3 LTCI, Télécom Paris, Institut Polytechnique de Paris, 91120 Palaiseau, France; 4 Department of Electronic and Computer Engineering, University of California – Santa Barbara, Santa Barbara, CA 93106 USA

**Keywords:** Diode lasers, Semiconductor lasers, Photonic devices

## Abstract

Stable laser emission with narrow linewidth is of critical importance in many applications, including coherent communications, LIDAR, and remote sensing. In this work, the physics underlying spectral narrowing of self-injection-locked on-chip lasers to Hz-level lasing linewidth is investigated using a composite-cavity structure. Heterogeneously integrated III–V/SiN lasers operating with quantum-dot and quantum-well active regions are analyzed with a focus on the effects of carrier quantum confinement. The intrinsic differences are associated with gain saturation and carrier-induced refractive index, which are directly connected with 0- and 2-dimensional carrier densities of states. Results from parametric studies are presented for tradeoffs involved with tailoring the linewidth, output power, and injection current for different device configurations. Though both quantum-well and quantum-dot devices show similar linewidth-narrowing capabilities, the former emits at a higher optical power in the self-injection-locked state, while the latter is more energy-efficient. Lastly, a multi-objective optimization analysis is provided to optimize the operation and design parameters. For the quantum-well laser, minimizing the number of quantum-well layers is found to decrease the threshold current without significantly reducing the output power. For the quantum-dot laser, increasing the quantum-dot layers or density in each layer increases the output power without significantly increasing the threshold current. These findings serve to guide more detailed parametric studies to produce timely results for engineering design.

## Abstract (web summary)

Investigation of the spectral narrowing physics of self-injection-locked on-chip lasers to Hz-level linewidth using a composite-cavity structure. Heterogeneously integrated III–V/SiN lasers operating with quantum-dot and quantum-well active regions are analyzed.

## Graphical abstract

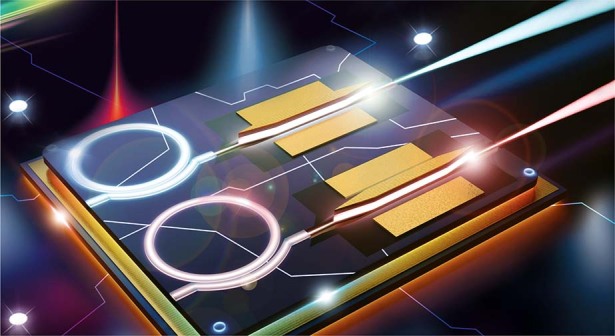

## Introduction

Quantum-confinement-based quantum-well (QW) and quantum-dot (QD) semiconductor laser diodes are the primary options for solid-state light sources, owing to their excellent characteristics of power efficiency, high-temperature operation, small form-factors, etc. While QWs have been adopted for years in commercialized products, QDs are of interest due to their zero-dimensional (0-D) density of states and atom-like degeneracy^1–4^. Since their first demonstration in 1994^5^, QD lasers have long surpassed their QW counterparts in terms of low transparency current density, high-temperature stability, and reduced sensitivity to external feedback and material defects^1,2,6,7^. The gain bandwidth of QDs can be engineered to emit a wide range of wavelengths throughout the near-infrared window by leveraging the inherent line-broadening effects^8,9^. Moreover, QDs are associated with a theoretical-zero linewidth enhancement factor (${\alpha }_{H}$). The much reduced ${\alpha }_{H}$ in QDs, compared to QWs, leads to larger damping rates (thus higher coherence collapse thresholds), less frequency chirping, ultrafast gain dynamics, suppressed filamentation, less vulnerability to noise and optical feedback, and easier frequency/phase stabilization in QD devices^1,10,11^.

That said, although homogeneous and inhomogeneous broadening phenomena in QDs are advantageous from the aforementioned viewpoints, they can adversely impact the purity of emission with comparatively high phase (or frequency) noise and, in turn, wide lasing intrinsic linewidths. For decades, several techniques have been investigated and employed to stabilize resonators. Compared to electronic means of laser stabilization, optical injection locking^12,13^ is a more compact and cost-effective alternative in which a stable narrow-linewidth emission is injected into the laser’s cavity to stabilize (‘lock’) its subsequent emissions^14,15^. Injection locking has been achieved and demonstrated in different forms, including the “master-slave” configuration via an external active pure seeding source^16^, the Pound–Drever–Hall technique via a stable cavity^17^, optical feedback injection (a.k.a. self-injection locking) via external optical circuits^18^, Bragg^19^ or holographic^20^ gratings in Littrow or Littman configurations, high-finesse Fabry–Perot (FP) cavities, etc^21^.

In an integrated photonic setting, self-injection locking can be realized by locking the emission of a laser diode to one or more eigenmodes of a high-*Q* integrated microring resonator. Since the first demonstration in 1998^22^ and based on the theoretical foundation^23–26^, high-*Q* crystalline and integrated microring resonators have been extensively utilized to achieve narrow linewidths^22,27–30^, low-noise photonic microwave oscillators^31^, and soliton comb generators^14,32^. In such a paradigm, the laser diode, along with the microring resonator, may be perceived as a composite resonator whose eigenmodes extend into free space. This composite resonator/free-space combination possesses its own resonance modes with additional benefits of selective mode suppression, power enhancement, and frequency stabilization against chirping effects and cavity-length fluctuation^33–35^. When a component of the laser’s emission is in resonance with one of the so-called whispering gallery modes of the microresonator, i.e., a composite-cavity mode, optical feedback injection occurs in the form of intracavity Rayleigh backscattering off the surface and volumetric nonidealities of the microresonator.

In microring resonators, the Silicon Nitride (SiN) platform has gained a great deal of popularity due to its comparatively low guiding losses, low thermo-optic coefficients, compatibility with the CMOS platform, being free from two-photon absorption in the telecom window, and high degree of Kerr nonlinearity for modulation and microcomb generation but simultaneously negligible Raman and Brillouin nonlinearities that limit the maximum allowed optical power^36–38^. With the aid of self-injection locking, hybrid integrated III–V lasers to chip-based SiN microresonators have been widely utilized to realize chip lasers and soliton microcomb sources^14,30,39–43^ with linewidths as narrow as 40 mHz^30^, competing with state-of-the-art fiber lasers. That said, heterogeneous integration of III–V lasers with SiN microresonators offers more stability, higher-volume production, compactness, and extra features with demonstrated performance far exceeding that of solitary III–V lasers grown on native platforms^36,44,45^. However, these demonstrations were performed on traditional off-the-shelf lasers. Only very recently, the first demonstration of integrating a high-Q microring resonator with a high quantum-confinement-based laser was reported^44^. In that work, an InP/Si multi-QW distributed feedback (DFB) laser was heterogeneously integrated with a high-Q SiN microring resonator on a monolithic Si substrate. This enabled a linewidth narrowing from 60 kHz to 25 Hz with self-injection locking with a 30-dB reduction in phase noise exhibited at a frequency offset of 300 kHz. That said, no experimental reports exist to date of an integrated QD laser with a SiN resonator in this context. The achieved linewidths at the tens of Hz level greatly surpass its intrinsic limitation of spectral impurity. This maturating of heterogeneous laser technology intensified the exploration of complex integrated III–V and Si/SiN optical configurations.

In this work, we perform a first-of-its-kind parametric analysis on self-injection locking in III–V/SiN composite cavities made up of QW- and QD lasers and ultrahigh-*Q* SiN resonators. As such complex quantum structures, we investigate the effects of active medium parameters, namely, the number of QW and QD layers and the average QD density per layer, over the optical and spectral performance characteristics. Unlike traditional theoretical works treating linewidth narrowing via external cavities in traditional lasers^23–26^ by focusing on the cavity-coupling dynamics, this work uniquely investigates linewidth narrowing enabled by their quantum-confined active mediums. This work analyzes and compares QW and QD active medium archetypes in terms of the major differences in injection locking and linewidth narrowing arising from carrier quantum confinement and associated density of state functions, gain saturation, and carrier-induced refractive index fluctuations (typically cast in terms of ${\alpha }_{H}$)^46,47^.

Lastly, we perform a multi-objective optimization study considering different laser performance criteria (objective functions). Previous reports exist in literature on optimizing the stability and locking range of self-injection locking via high-Q resonators focusing on parameters concerning the injection-locking mechanism itself^48,49^, such as the coupling strength, roundtrip time, frequency detuning, and pump coupling efficiency. Instead, in this work, we focus on the design parameters of the QW/QD active region in addition to the injection current density as optimization variables to optimize the laser performance in the locked regime in terms of linewidth-narrowing and power efficiency. The aim of this work is to uncover the effects of growth design parameters of such complex systems and facilitate design and engineering decision-making in the future while shedding light on underlying physics involved in the observed effects.

## Results

**Fig. 1:**
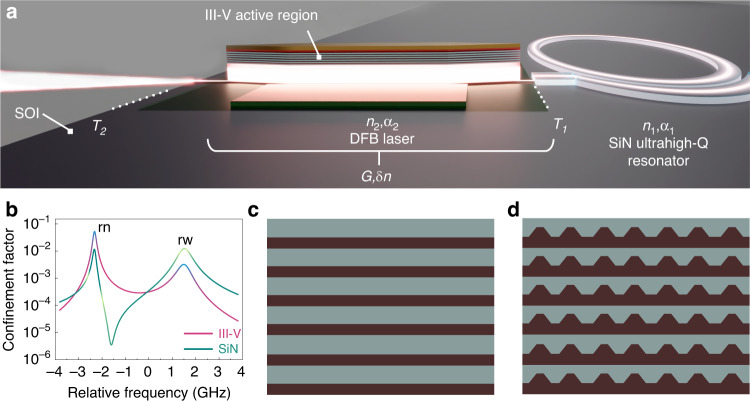
Basic III–V/SiN coupled-cavity configuration used in the calculations. **a** Schematic of the III–V/SiN coupled-cavity structure. Input to the laser theory and connection to experimental devices is through the passive cavity resonances and *Q* factors, as well as an effective coupling between cavities ${T}_{1}$. The gain and carrier-induced refractive index ($G$ and $\delta n$, respectively) are calculated from the laser theory. **b** Two resonances of the coupled system in the III–V and SiN cavities. Each composite mode contributes to a point in the resonances. Together, they produce the finite-width (Fox–Li) quasi-mode resonances caused by outcoupling. The resonances are labeled rn and rw for resonant narrow and resonant wide. **c**, **d** Illustrations of the two active region structures of QWs (**c**) and QDs (**d**)

To investigate the differences between the QD and QW gain regions in terms of linewidth narrowing resulting from self-injection locking in the coupled III–V/SiN compound cavity, a generic 1-d compound cavity structure was utilized. As depicted in Fig. 1a, two coupled resonators with cavity-quality factors ${Q}_{1}$ and ${Q}_{2}$ represent the III–V and SiN sections, respectively. Both resonators are coupled via an effective coupling transmission of ${T}_{1}=0.01$ and an outcoupling transmission of ${T}_{2}=0.02$. Meanwhile, the cavity length and refractive index of the SiN resonator were set as ${L}_{1}=4\,{\rm{mm}}$ and ${n}_{1}=2.1$, respectively, and as ${L}_{2}=600$ μm and ${n}_{2}=3.6$ for the III–V section, respectively. The results of this investigation are attained by following the formulated theoretical analysis we laid in ref. ^50^ to firstly obtain the eigenmodes of the passive compound cavity, followed by numerically solving for the time evolution of the intensity and frequency of each eigenmode due to the III–V active medium. Anchoring of the theoretical model is provided by measurements on QW and QD DFB lasers presented in ref. ^51^ and ref. ^52^, respectively.

Figure 1b shows the calculated resonance profile for each of the III–V and SiN cavities in resonance with each other, showing two closely spaced composite-cavity resonances, i.e., resonant narrow (rn) and resonant wide (rw), with splitting determined by ${T}_{1}$. Each resonance is composed of multiple composite-cavity modes. The linewidths and Q factors of the uncoupled cavities are 550 MHz and 3.9 × 10^5^ for the III–V cavity and 60 MHz and 3.6 × 10^6^ for the SiN cavity. Figure 1c, d depicts a sketch exemplifying an active region comprised of 6 layers of QWs and QDs, respectively. It was assumed that an intracavity filter, such as a distributed Bragg reflector (DBR), enabled a single quasi-Fox–Li mode operation at the rn resonance. First, the composite-cavity mode frequencies and eigenfunctions were calculated. For the rn resonance, the resolution of the Fox–Li mode required a few hundred composite-cavity modes. The composite-cavity eigenfunctions were used to compute the mode confinement and overlap factors. Then, mode intensities and lasing frequencies were computed.

### Integrated III–V/SiN QW laser

**Fig. 2:**
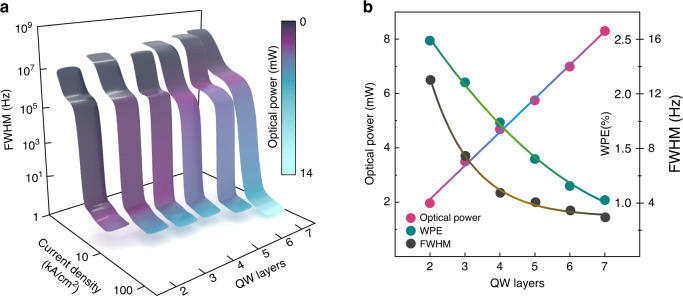
Linewidth and lasing characteristics of the integrated III–V/SiN QW laser. **a** Linewidth and lasing characteristics of the integrated III–V/SiN QW laser as a function of the injected current density and number of QW layers. **b** The optical power and FWHM at a current density injection of 3*J*th for different numbers of QW layers alongside the WPE

Figure 2a shows the steady-state solution for the full-width-at-half-maximum (FWHM) of the laser linewidth as a function of the injection current densities ($$J)$$ and different numbers of QW layers. The corresponding output optical power at each operation point on the surface is color-coded. At any fixed $$J$$, the output power increases with the number of QW layers due to the increased active medium volume, leading to larger differential gain and smaller quasi-Fermi-level separation^53^. To better visualize the trends with increasing the QW layers, Fig. 2b depicts the device’s optical power and FWHM at $$J=3{J}{\rm{th}}$$ along with the wall-plug efficiency (WPE), which is defined as the total output optical power divided by the total input electrical power. The optical power at $$J=3{J}{\rm{th}}$$ shows a nearly-linear increase with the QW layers at a rate of 0.85. On the other hand, the WPE surprisingly decreased from ~2.5% to ~1% as the number of QW layers increased from 2 to 7. The efficiency degradation is indicative of increased QW losses and can adversely impact the device’s performance^53,54^. One of the major reasons behind the observed low WPEs is the low outcoupling transmission $${T}_{2}$$, which limited the external quantum efficiency, and in turn, the WPE. We adopted such a low $${T}_{2}$$, i.e. a high effective reflectivity $${R}_{2}$$, to account for the coupling mechanism between the III–V active medium and Si waveguide as well as to focus on the intracavity dynamics and maximize the interactions and observed effects of linewidth narrowing inside the coupled cavity. In practice, however, the actual value of $${T}_{2}$$ can be increased, along with the external quantum efficiency and WPE, by tuning the design of the DFB cavity, as well as optimizing the coupling mechanism, e.g., coupling tapers or photonic crystals, between the III–V active medium and Si waveguide. The actual experimental device may also suffer from effects stemming from the poor transport of carriers across multi-QWs. Carrier distribution nonuniformity can lead to dissimilar populations of carriers in different wells; each is independently in equilibrium with its own quasi-Fermi level^55^.

From a spectral perspective, the linewidth narrowing in an integrated III–V/SiN laser can be observed in Fig. 2a to take place in three stages: Immediately pass the lasing threshold from gain clamping, according to the Schawlow-Townes description; an intermediate region, where locking to the high-*Q* SiN passive resonator begins to take place; and at sufficiently high injection current, total locking of III–V laser and SiN resonator is achieved, leading to significant linewidth reduction. However, it can also be observed from Fig. 2a that the locking mechanism becomes more demanding and occurs at higher currents with increased QW layers. In other words, as the active medium’s volume increases, locking conditions become more stringent due to the elevated injection ratio requirement.

It is worth pointing out that the locked spontaneous-emission-limited linewidths predicted here are within an order of magnitude of the range experimentally measured for the integrated InP/Si QW lasers reported in ref. ^44^. With that said, in terms of absolute FWHM, more QW layers resulted in narrower linewidths for any given $J$. Specifically, increasing the number of QW layers from 2 to 4 reduced the linewidth FWHM from 13 to 4.5 Hz. We ascribe this to the appreciable reduction in ${\alpha }_{H}$ due to the increased differential gain ($\partial G/\partial {n}_{c}$), owing to the increased carrier density of states at the band edge. ${\alpha }_{H}$ quantifies the amplitude-phase fluctuation coupling of semiconductor lasers under current injections that alter the carrier distribution. It is a key parameter in semiconductor lasers that characterizes not only the linewidth broadening effects but also many other aspects of the laser dynamics, such as frequency chirp, optical feedback effects, the formation of optical frequency combs, and the self-injection-locking detuning. In the late 1960s, the concept of phase-amplitude coupling in semiconductor lasers was observed^56,57^, which was later quantified by Henry in his renowned paper^26^ as ${\alpha }_{H}$. It was defined as the ratio between the derivatives of the real and imaginary parts of the susceptibility $\chi$, which in essence are the derivatives (with respect to carrier density ${n}_{c}$) of the effective refractive index $({\rm{\delta }}n)$ and the optical gain of the medium $(G)$, respectively:

1 $${\alpha }_{\rm{H}}=\frac{\partial \chi ^{\prime} /\partial {n}_{c}}{\partial \chi ^{\prime\prime}/\partial {n}_{c}}=-\frac{4\pi }{\lambda }\cdot \frac{\partial {\rm{\delta }}n/\partial {n}_{c}}{\partial G/\partial {n}_{c}}$$

where the optical susceptibility is $\chi =\chi ^{\prime} +i\chi ^{\prime\prime}$, the intensity gain is $G=-K\chi ^{\prime\prime}$, the carrier-induced refractive index is $\delta n=n\chi ^{\prime} /2$, $K=2\pi /n\lambda$ is the wavevector, $n$ is the background refractive index, and $\lambda$ is the laser wavelength. The linewidth enhancement factor may be used to gauge the extent of carrier-induced refractive index influence on semiconductor laser properties, such as linewidth and sensitivity to optical feedback. In laser theory, there is a linear as well as nonlinear contributions to the carrier-induced refractive index change, *viz*., frequency pulling and pushing, respectively^58^. As shown in Eq. 1, ${\alpha }_{H}$ is typically evaluated with the linear susceptibility, i.e., assuming only total carrier density dependence. In this case, derivations of quantitative relations between ${\alpha }_{H}$ and laser spectral properties are possible because the linear assumption is valid under quasi-equilibrium condition, and simple Class B rate equations adequately describe the laser behavior. However, we found in the stimulations of III–V/SiN lasers operating close to spontaneous-emission-limited linewidth, nonlinear effects become appreciable, resulting in intracavity laser intensity dependence in ${\alpha }_{H}$. An indication of the appreciable nonlinear contributions is the rollover in an L–I curve. These nonlinearities may be described by the gain compression and frequency pushing contributions. Their presence sufficiently complicates the multimode laser field equations that we are presently unable to derive expressions for relating ${\alpha }_{H}$ to, for example, the laser linewidth. Nevertheless, the improved linewidth performance with more added layers comes from reducing the lasing carrier density, which contributes to a higher differential gain^59^, and hence a larger denominator in Eq. 1. The increase in $\partial G/\partial {n}_{c}$ (dominator of Eq. 1) with more QW layers is reflected in the higher rate of power increase in Fig. 2a with respect to injected carriers as the number of QW layers increases. In addition, higher intracavity laser intensity increases the frequency pushing contribution, counteracting the frequency pulling (linear) contribution, leading to a smaller numerator. Equally, if not more, important is that the increased intracavity laser fields also increase the frequency locking term in our laser equations, thus allowing the final step towards Hz or sub-Hz linewidth.

That said, when the number of QW layers was increased beyond four, diminishing returns in the FHWM reduction were observed with the lowest FWHM of ~3 Hz with 7 QW layers, compared to the 4.5 Hz with 4 QW (see Fig. 2b). Such behavior suggests that a tradeoff is in play in the enhancement of the linewidth between the carrier-induced change in refractive index and the differential gain. This hinders the attainable benefits of increasing the number of QW layers beyond a certain point. In a later part of this manuscript, we perform a multi-objective optimization investigation considering different laser performance criteria (objective functions).

### Integrated III–V/SiN QD laser

**Fig. 3:**
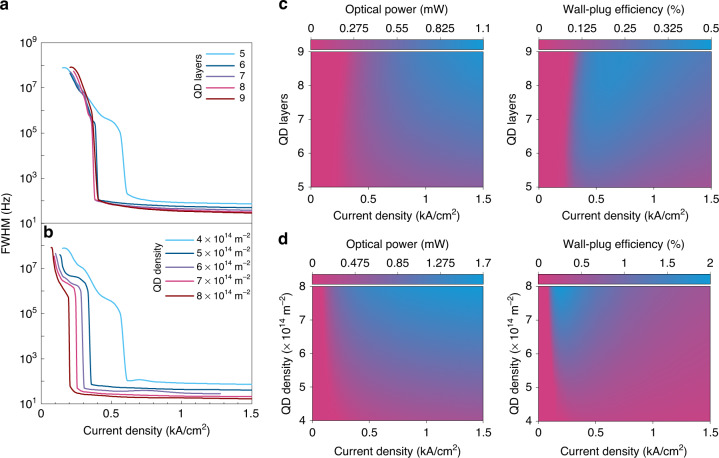
Linewidth and lasing characteristics of the integrated III–V/SiN QD laser. Linewidth FWHM of the III–V/SiN QD laser as a function of the injection current density for different QD layers (**a**) and QD densities (**b**). Colormaps of the output power (left) and wall-plug efficiency (right) as functions of the QD layers (**c**) and QD density (**d**)

Next, a similar examination was followed on the performance of the integrated III–V/SiN QD laser with two design parameters, *viz*., the number of QD layers in the active medium and the average dot density per layer. With a fixed dot density of 4 × 10^14^ m^−2^, Fig. 3a shows the change in the linewidth FWHM with the current density as a function of the number of QD layers. Similarly, at a fixed number of QD layers of five, Fig. 3b shows the change in the linewidth FWHM with the current density as a function of the QD density. Like the QW device, increasing the QD layers or dot density reduced the linewidth FWHM. For instance, increasing the number of QD layers from 5 to 6 appreciably reduced the FWHM (at $J=3{J}{\rm{th}}$) from ~200 Hz to ~75 Hz (−62.5%), while increasing the QD layers beyond that point resulted in diminishing returns, i.e., narrowing from ~75 to ~45 Hz at the cost of the extra 4 QD layers. Similarly, the linewidth FWHM linearly decreased from ~97 to ~30 Hz (−70% decline at a rate of $-$17 × 10^−14^ Hz/m^−2^), when doubling the dot density from 4 × 10^14^ to 8 × 10^14^ m^−2^. In both QW and QD devices, after full locking, the linewidth decreases with intracavity intensity, which increases with the number of layers for both QW and QD devices.

On the other hand, contrary to the observed trend in the QW device, attainment of locking becomes easier, i.e., taking place at a lower current density, with increased QD layers/density (notice the point of steep linewidth reduction in Fig. 3a, b). We attribute the difference in the linewidth performance between the QW and QD lasers to the difference in their ${\alpha }_{H}$, being nearly zero in the latter. This is a result of the frequency pulling in QWs being dependent on carrier density (due to their asymmetric carrier density distribution) while being independent of carrier density in QDs (due to their symmetric inhomogeneously broadened carrier density distribution).

QDs are typically grown via the Stranski–Krastanov mode. The resulting inherent size dispersive nature gives rise to homogeneous and inhomogeneous broadening phenomena. Therefore, in terms of absolute linewidth, QDs are sometimes associated with broader linewidths compared to the less quantum-confined nanostructures, e.g., QWs. However, self-injection-locking can drastically reduce their linewidth FWHM, owing mainly to their near-zero ${\alpha }_{H}$ – a distinctive feature of QDs. Via self-injection locking, the threshold current of the locked mode to which the bias current contributes the most is decreased. Meanwhile, the modal gain of the other modes is reduced due to the decreased carrier occupation probabilities^60^. ${\alpha }_{H}$ strongly depends on the occupation probability of carriers in the individual QDs, such that the lower the occupation probability, the lower ${\alpha }_{H}$ is^11^. In other words, increasing the QD layers or density translates to a lower carrier occupation probability per QD group as the total number of dots increases. Consequently, this leads to a smaller ${\alpha }_{H}$, and ultimately a stronger linewidth reduction and easier attainment of injection locking.

On another front, in terms of power and efficiency, Fig. 3c shows 2D colormaps of the output power (left) and wall-plug efficiency (right) of the QD device as functions of the injection current density and the number of layers. Figure 3d shows the same, albeit for different QD densities, instead of number of layers. Much like the case of the QW laser, either increasing the number of QD layers or dot density per layer resulted in higher optical powers for any fixed $J$ (any vertical slice of Fig. 3c (left) or Fig. 3d (left), which can be attributed to the increased effective volume of the active medium^61,62^. Furthermore, any horizontal slice of Fig. 3c (left) or Fig. 3d (left) gives the L–I relation for that particular number of QD layers/density. That said, Fig. 3c (right) and Fig. 3d (right) show that the efficiency increases with increasing the QD layers/density for any fixed current density (any vertical slice of Fig. 3c and d). This is in stark contrast to the observed reduction in efficiency with more layers in the case of the QW counterpart. In addition, any horizontal slice of Fig. 3c (right) or Fig. 3d (right) also indicates that the efficiency is maximum right after the locking point and starts to decay as the current increases (L–I rollover). Like bulk and QW structures, QDs suffer from defect losses, yet to a much lesser degree due to their strong confinement associated with their 0-D density of states.

### Parameter optimization and engineering considerations

**Fig. 4:**
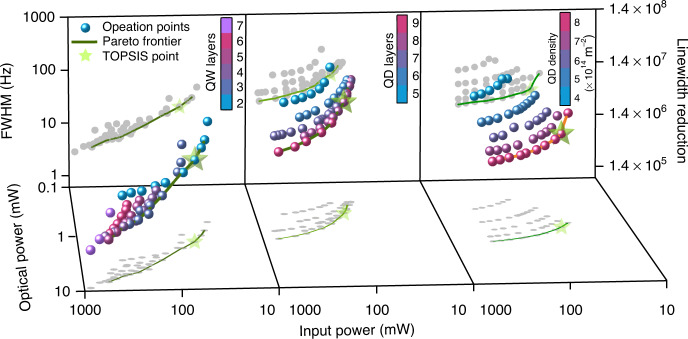
Optimization of the integrated III–V/SiN QD and QW lasers showing the 4D design space, the Pareto frontier, and the TOPSIS optimal points for different QW layers, in the case of the integrated III–V/SiN QW laser, and for different QD layers/densities for the integrated III–V/SiN QD laser

The complexity of the optimal operation point and design parameters have been analyzed in the integrated III–V/SiN QW and QD lasers, with several tradeoffs in play between competing parameters with a few conflicting performance criteria. In the following, we perform a multi-objective optimization of the performance parameters of the III–V/SiN QW and QD lasers, taking into account four objective functions (criteria) with respect to the number of QW/QD layers, QD surface density per layer, and injection current density as optimization variables. In this optimization process, the adopted objective functions are: minimizing the input power, maximizing the output optical power, maximizing the wall-plug efficiency, and minimizing the intrinsic linewidth FWHM (maximizing the linewidth narrowing due to self-injection locking). Figure 4 shows the 4-dimensional design space of the III–V/SiN QW and QD lasers showing the FWHM and input and output powers of each point. Furthermore, the shown colormaps indicate the number of layers (or QD density) of each point. The projections of each point on the XY and XZ planes are also depicted in gray for clarity. It is worth mentioning that only points beyond the locking point are considered here. Firstly, a genetic algorithm was employed to obtain the Pareto frontier for each device (traced by the green curves in Fig. 4) consisting of a set of Pareto optimality points, defined as the set of points where no performance criterion can objectively be made better off. In other words, for each point outside of the Pareto frontier, there exists at least one point on the Pareto frontier that is objectively better in at least one performance criterion. Ultimately, the Pareto frontier serves as a guide to which points are viable to consider as a potential optimal point. Rather than crudely picking a set of optimal points manually, the genetic algorithm serves a systematic way to achieve that by quantifying where each point stands in terms of the considered objective functions and then highlighting a border of objectively superior points (i.e., the Pareto frontier). The Pareto frontier provides the most feasible alternatives to the device designer, who can validly pick any point along it based on their desired requirements and priorities, extending beyond the investigated sample points.

Thereafter, we followed with the Technique for Order of Preference by Similarity to Ideal Solution (TOPSIS), a multi-criteria decision-making algorithm developed by Hwang and Yoon^63^. In the realm of integrated photonics, power consumption remains an essential attribute in order to satiate the demand for reduced switching and operation power consumption in the tele- and data-com industries, which gave rise to Si photonics in the first place^64^. Meanwhile, although the intrinsic linewidth is of great significance, all the considered points here are already within the locking range of the III–V/SiN coupled cavity with substantially low FWHM for all intents and purposes. Therefore, minimizing power consumption was adopted as the most pressing concern.

Following this, the obtained optimal operation point for each case is shown in Fig. 4. For the integrated III–V/SiN QW laser, the optimal point was found to be that of an input power of ~54 mW, an output optical power of ~1.4 mW, a linewidth FWHM of ~18 Hz, and a wall-plug efficiency of ~2.6%. This point is obtained with 2 QW layers and an injection current density of ~2 kA/cm^2^. Considering the associated power consumption, minimizing the number of QW layers and maintaining moderate current density injection are recommended.

Conversely, the optimal point for the integrated III–V/SiN QD laser was found to be manifested with 8 QD layers and an injection current density of ~0.5 kA/cm^2^ (~52 mW input power), resulting in an output optical power of ~0.43 mW and a linewidth FWHM of ~67 Hz. Similarly, in terms of the dot density, the TOPSIS optimal point was found to be of an output optical power of ~0.96 mW and a linewidth FWHM of ~30 Hz, obtained with 8 × 10^−14^ m^−2^ QD density and an injection current density of ~0.25 kA/cm^2^. In other words, contrary to the III–V/SiN QW case, the analysis shows that maximizing the number of QD layers or QD density yields optimal performance parameters when injected with low current density.

The corresponding input powers of ~54 and ~52 mW would translate into a switching energy consumption of ~5.4 and ~5.2 pJ/bit, given a 10 Gbit/s direct modulation in an ON–OFF Keying (OOK) modulation format. This was estimated based on the fact that 54(52) mW means 5.4(5.2) mJ of input energy is distributed among 10 billion bits every second.

## Discussion

**Fig. 5:**
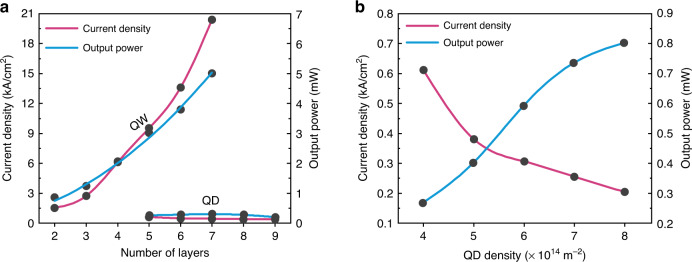
Comparison of the lock conditions of the III–V/SiN QW and QD lasers. Comparison of the lock conditions, where the steep reduction in the intrinsic linewidth occurs, in terms of the required injection current density and corresponding output power as functions of the number of layers (**a**) for the integrated III–V/SiN QW and QD lasers and as functions of the QD density for the QD laser (**b**)

In this section, we compare the locking phenomenon in both integrated III–V/SiN QW and QD devices. To that end, Fig. 5a illustrates the effect of increasing the number of layers in both devices on the locking threshold condition, i.e., the onset of self-injection locking, in terms of the required current density, alongside the corresponding output optical power. Similarly, Fig. 5b shows the effect of increasing the QD density while fixing the number of QD layers to five. At first glance, the selling point of each device becomes rather apparent. On the one hand, the coupled integrated III–V/SiN QW laser offers more optical power, which only grows larger with more added QW layers. However, this is achieved at the expense of drastically increased current density and input power requirements, adversely diminishing the quantum and wall-plug efficiency. This is where the integrated III–V/SiN QD laser shines. While it indeed lags behind the QW counterpart in terms of absolute output optical power, injection locking and the steep reduction in the linewidth of the integrated III–V/SiN QD laser take place at a much lower current density (and input power) with increased QD layers or QD density. Improving the efficiency and reducing locking requirements can therefore be achieved with increased QD density or layers, unlike the QW counterpart. Nonetheless, although increasing either the QD layers or QD density yielded similar trends in the examined performance parameters, these trends were much stronger with increasing the dot density per layer. We attribute this to the substantially higher QD packing density (dots per unit volume) in the former approach. This is because increasing the QD layers entails more QW layers, in which each QD layer is embedded, in addition to capping/barrier layers. This, in turn, would drastically dilute the active medium’s dot packing density compared to increasing the QD surface density per layer, allowing for better realization and utilization of the QD attractive features.

The results also indicate an important point. Typically, standalone freerunning QW lasers suffer from high ${\alpha }_{H}$ that are further elevated with injection current. At the same time, QD lasers suffer from wide extrinsic linewidths. However, introducing the SiN microring resonator here enabled both integrated III–V/SiN devices—when locked—to circumvent these linewidth-related drawbacks, as both devices are able to achieve similar linewidth performance, even at higher current injections. Yet, the QD-based device does so more efficiently, while the QW emits higher optical powers.

## Methods

To carry out the modal intensities and lasing frequencies within the compound cavity, we followed the formulated theoretical analysis given in ref. ^50^. The theoretical approach includes (a) multimode laser interaction to treat mode competition and wave mixing, (b) quantum-optical contributions from spontaneous emission and (c) composite laser/free-space eigenmodes to describe outcoupling and coupling among components within an extended cavity. Table 1 depicts the common composite-cavity structure and gain medium parameters of the integrated III–V/SiN QD and QW lasers. The gain medium parameters were extracted from the QW and QD DFB lasers presented in ref. ^51^ and ref. ^52^, respectively. The fact that the gain medium parameters of both devices are identical is predicted by quantum kinetic calculations, owing to the balance among different Coulomb correlation contributions^56,65,66^. It also indicates that the obtained results in this analysis are exclusively based on the difference between 0-D and 2-D carrier densities of states.

**Table 1:** Parameters for integrated III–V/SiN QD and QW lasers

Region	Parameter	Symbol	QD	QW	
Composite cavity	QW height	*h*_qw_ (nm)	8 nm		
	Waveguide height	*h*_wg_ (μm)	0.2 μm		
	Stripe width	*w* (μm)	4.9 μm		
	SiN cavity Q factor	*Q*_SiN_	3.6 × 10^6^		
	III–V cavity Q factor	*Q*_III–V_	3.9 × 10^5^		
	Spontaneous-emission factor	*β*	0.003		
	QW height	*h*_qw_ (nm)	8 nm		
Gain medium	Dephasing rate	γ (s^−1^)	10^12^ s^−1^		
	Population relaxation rate	γ_ab_ (s^−1^)	10^12^ s^−1^		
	Defect loss rate	*γ*_nr_ (s^−1^)	10^9^ s^−1^		
	Bimolecular recombination rate	B_3d_ (m^3^ s^−1^)	10^−16^ m^3^ s^−1^		
	Lateral confinement factor	Γ_xy_	0.20		0.28
	Inhomogeneous broadening	Δ_inh_ (meV)	10		–

Depending on various parameters, the performance of presently fabricated devices may differ from what theoretical analyses predict because of extrinsic factors. This is particularly true when considering exceedingly complex structures such as the investigated III–V /SiN QD integrated lasers, whose fabrication has proven to be extremely challenging. In that regard, the aim of this work is to guide ongoing experiments by modeling device behavior over a broader parameter space that is experimentally practical. For that goal, top-level device models depending on phenomenological input parameters, as is often the case with basic rate equations, are inadequate. Instead, we adopted an approach that directly connects microscopically (i.e., at the level of electrons and holes) to band structure and optical design, where there is a high degree of practical control and predictability thanks to the considerable progress in growth technology and to the highly matured semiconductor and silicon fabrication techniques.
